# S-Shaped Wide Excision with Primary Closure for Extensive Chronic Pilonidal Sinus Disease

**DOI:** 10.1155/2014/451869

**Published:** 2014-06-02

**Authors:** Kerem Karaman, Safak Ozturk, Cem Tugmen, Eyup Kebapcı, Sait Murat Dogan, Mutlu Unver, Mustafa Olmez, Cengiz Aydin

**Affiliations:** ^1^Department of General Surgery, Faculty of Medicine, Sakarya University, 54055 Sakarya, Turkey; ^2^Department of General Surgery Clinic, Tepecik Teaching and Research Hospital, Izmir, Turkey; ^3^Department of General Surgery Clinic, Bozyaka State Hospital, Izmir, Turkey

## Abstract

*Background*. The management of complex pilonidal sinus disease (PSD) with multiple pits on and beside the natal cleft is variable, contentious, and problematic. Wide excision of the sinus and reconstruction of the defect using different flap techniques have become more popular in recent years. *Case Report*. We report a case with a complex chronic PSD to which we applied primary closure after S-shaped wide excision. The patient's postoperative course was uneventful, and at the end of one-year followup he is now disease-free and comes for routine checkups. *Conclusion*. The simplicity of the technique and the promising results support the applicability of the S-shaped wide excision in chronic bilaterally extended large PSDs. Further studies entailing large patient populations are needed to reach a definite conclusion.

## 1. Introduction

PSD is a debilitating, painful, chronic inflammatory disease that is caused by penetration of the skin by loose hair. It generally presents as cyst, abscess, or one or more discharging painful sinus tracts in the upper part of the natal cleft [1].

PSD is now generally believed to be an acquired condition. The depth of the intergluteal sulcus, the number of loose hairs, lacerations in the skin due to trauma and erosion due to moisture and friction, large pores, and the weakness of the skin in the midline can facilitate hair entrance [2, 3]. The management of complex PDS with multiple pits on and beside the natal cleft is variable, contentious, and problematic. Principles of treatment require eradication of the sinus tract, complete healing of the overlying skin, and prevention of recurrence. Wide excision of the sinus and reconstruction of the defect using different flap techniques have become more popular in recent years [4, 5]. We report on a case with a complex chronic PSD to which we applied primary closure after an S-shaped wide excision.

## 2. Case Report

**Figure 1:**
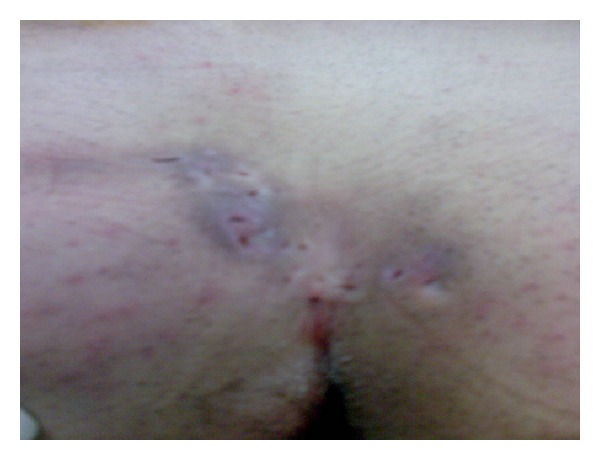
Demonstration of the complex pilonidal sinus disease with multiple pits on and beside the natal cleft.

**Figure 2:**
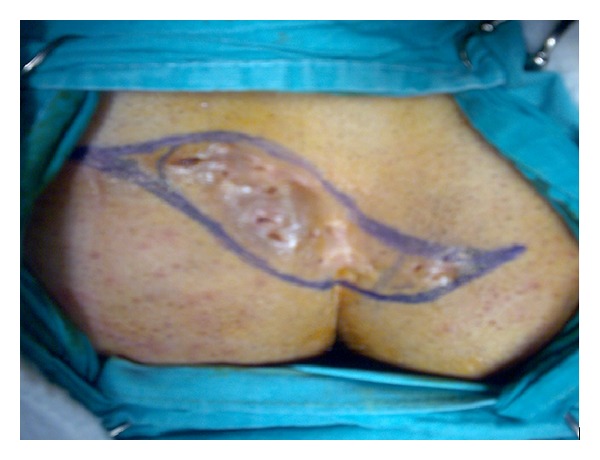
Landmarks of the incision.

**Figure 3:**
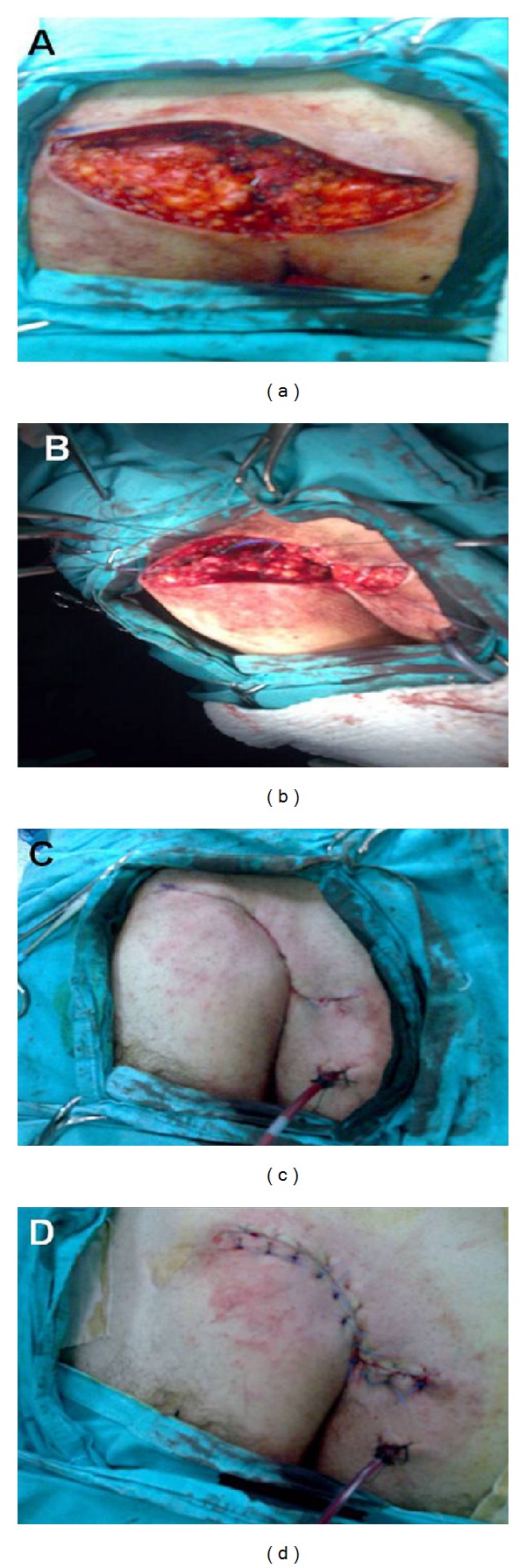
Demonstration of the patient's operation: (a) resection of the pilonidal cyst and the sinus tracts with the underlying subcutaneous tissue and fat; (b) a relaxation incision was made in the fascial layers of the wound edges, by incising the gluteus muscle fascia vertically on either side, which allowed rotation of the skin and the underlying tissue for tension-free primary closure; (c) both wound edges were approximated subcutaneously with deep interrupted sutures using 0 polyglactin; (d) a closed suction drain was inserted in the cavity.

**Figure 4:**
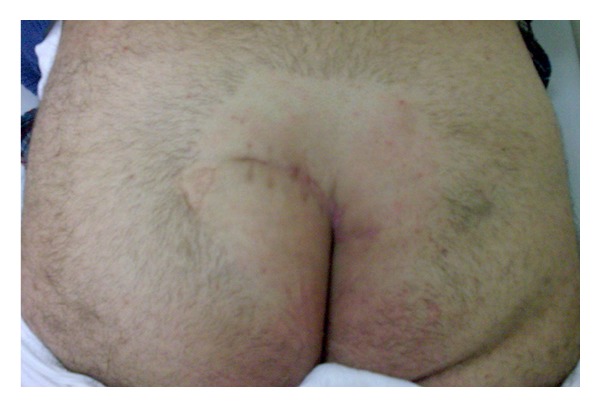
Postoperative appearance of the operative field at the end of one-year followup.

A 41-year-old male patient was admitted with complaints of continuous abscess and purulent discharge in the sacrococcygeal region of more than 5 years in duration. His physical examination revealed a chronic PSD, including hair follicles with multiple pits in the natal cleft as well as on both sides of the gluteal cutaneous tissue (Figure 1). It was decided primarily to perform debridement of all granulation tissue associated with the pilonidal cyst. The site of the operation (gluteal and sacral region) was shaved on the day of surgery. A single dose of 1 gr cephalosporin (Sefazol, Mustafa Nevzat Ilac Sanayi AS, Istanbul, Turkey) was administered for prophylaxis 30 minutes before surgery. The operation was performed with the patient in the prone jackknife position under spinal anesthesia. The buttocks were spread and taped to make the sinus holes and the intergluteal sulcus more visible. The area to be incised was marked (Figure 2). Methylene blue was used prior to skin incision to fill the cystic cavity, to find fistulous connections, and to determine safe resection margins. Two parallel S-shaped incisions, encompassing the wound area that extended on both sides of the gluteal regions and coalesced in their origin and terminal endpoints, were performed. After resection of the pilonidal cyst and the sinus tracts (Figure 3(a)), a relaxation incision was made by incising the gluteus muscle fascia vertically on either side, which allowed rotation of the skin and the underlying tissue for tension-free primary closure. A closed suction drain was inserted in the cavity. Both wound edges were approximated subcutaneously with deep interrupted sutures using 0 polyglactin (Vicryl, Ethicon, Johnson & Johnson Medical, Somerville, NJ, USA) (Figures 3(b) and 3(c)), and the skin was closed by mattress sutures with 3/0 polypropylene (Prolene, Ethicon, Johnson & Johnson Medical, Somerville, NJ, USA) (Figure 3(d)). The patient's postoperative course was uneventful and he was discharged on the 5th postoperative day after removing the drain, and skin suture materials were taken out on the 14th postoperative day. The histopathological diagnosis was chronic pilonidal cyst. At the end of one-year followup, the patient is now disease-free and comes for routine checkups (Figure 4).

## 3. Discussion

Numerous techniques and modifications are in use for the treatment and prevention of PSD. However, a consensus on the optimal treatment of PSD has never been formed. The most common treatment approach is the excision of the cyst cavity. The traditional treatment modalities, either leaving the wound open to heal by secondary intention or the primary closure, are the most commonly used techniques worldwide [6]. Neither method is flawless: whereas PSD wounds heal more quickly after primary closure, the risk of sinus recurrence is higher than with open healing. However, no significant difference in the rate of healing has been found between the two approaches over the long term [7, 8]. A clear benefit in terms of recurrence has, however, been seen when using off-midline closure compared to midline closure [9, 10].

Simple excision with primary closure not only leads to faster convalescence, but also results in a midline scar in a persistent deep natal cleft, potentially leading to high recurrence rates. Therefore, flattening the natal cleft is recommended, which decreases the generation of sweat and friction caused by buttock movement, skin maceration, and debris accumulation [11]. In order to avoid median recurrences and flatten the natal cleft, numerous operative techniques have been developed such as the Karydakis technique; the Bascom procedure; rhomboid excision with Limberg flap closure; Z-plasty or rotation flap [2, 12–14]. The standard rhomboid flap application seems to be the most effective technique with the lowest recurrence rates. However, healing of the lower end of the flap is problematic; indeed, this area is the dampest and the dirtiest part of the body where maceration and chronic wound formation may develop and so delay recovery. To prevent this undesirable outcome, the modified Limberg flap reconstruction was designed, where the rhomboid excision is made asymmetrically to place the lower end of the flap lateral to the intergluteal sulcus [15].

**Figure 5:**
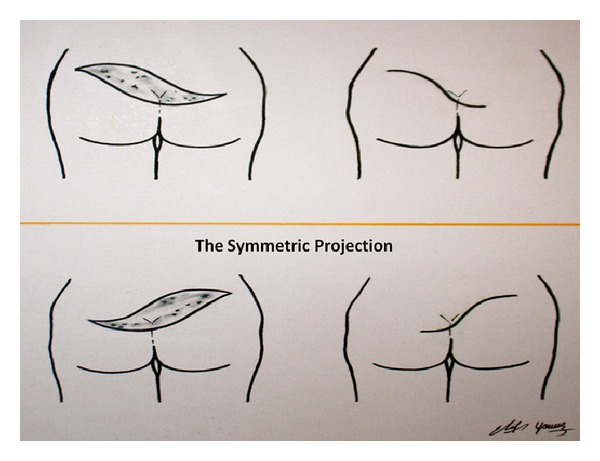
Illustration of the primary closure technique after S-shaped wide excision.

However, it is difficult to manage complex PSDs that extend to gluteal regions bilaterally due to the broad area that must be reconstructed after a wide resection. In our case, we chose to use two parallel S-shaped skin incisions that encircle the wound area and merge at top and bottom endpoints (Figure 5). To facilitate tension-free primary closure, relaxation incisions in the fascial layers on both sides of the wound edges were also necessary.

Some similar techniques do exist in the literature. In the study by Yildar et al., a second skin incision is performed after an S-type excision for flap reconstruction [16]. Krand and coworkers applied gluteus maximus fascia advancing flap reconstruction—as they stated—on patients with PSD who did not have extensive gluteal involvement [17]. On the other hand, in the present case, S-shaped incisions were used due to an extensive involvement and the S-shaped incisions were much sharper than an oblique skin incision. A similar technique was also used in a clinical study by Kim et al. in 17 patients [18]. However they defined the technique as S-plasty. Our technique differs from theirs, as we used multiple subcutaneous sutures—instead of mattress sutures—for closure of the death space between the wound edges and used mattress sutures only for skin closure. This should reduce tension on the skin edges and prevent ischemic events leading to wound infection.

Although the usefulness of drainage in flap reconstructions is controversial, we advise a closed suction drain in wide excisions [19–22]. Laser depilation is recommended as an adjunct to surgery with the aim of decreasing the change of recurrence [23, 24]. One disadvantage of the present technique may be a predisposition to wound infection in the lower part of the flap, at the point where it passes through the natal cleft. However, this risk is also present in the standard Limberg closure, where the lower end of the flap ends in the intergluteal sulcus.

In conclusion, the simplicity of the technique and the promising results support the applicability of the primary closure after S-shaped wide excision in chronic bilaterally extended large PSDs. Further studies consisting of large patient populations are needed to reach a definite conclusion.
